# Glycemic and nonglycemic mechanisms of congenital malformations in hyperglycemic pregnancies: a narrative review

**DOI:** 10.20945/2359-3997000000521

**Published:** 2022-10-03

**Authors:** Carlos Antonio Negrato, Paulo Rubens Marques, Heloisa Barreto Leite, Carolina Naomi Torigoe, Beatriz Fernandes Silva, Kessy Costa, Júlia Marchatto Kamei, Cárian Leoz Zampa, Ana Clara Rocha Gomes Toni, Izabella Carolina Gomes Santana Pereira, Gabryel Luz Heinzelmann, Lenita Zajdenverg

**Affiliations:** 1 Universidade de São Paulo Bauru Faculdade de Odontologia de Bauru SP Brasil Faculdade de Odontologia de Bauru, Universidade de São Paulo Bauru, SP, Brasil

**Keywords:** Congenital malformations, hyperglycemia and maternal diabetes

## Abstract

Congenital malformations are more frequently found among children born to mothers with diabetes than in the background population. There are several complex mechanisms involved in the development of congenital malformations in the offspring of mothers with hyperglycemia, such as the overexpression of glucose transporters (GLUTs) 1 and 2, the increased activity of the hexosamine biosynthetic pathway and the reduced expression of the PAX3 gene with a consequent increase in p53 protein expression. These alterations can lead to increased glucose and free radical concentrations in the embryo, thus promoting the process of apoptosis and causing malformation. The most frequent malformations found in the offspring of mothers with diabetes are heart and neural tube defects, urinary tract and kidney malformations, and cleft lip with or without cleft palate. Strict glycemic control should be obtained before and during pregnancy, aiming to avoid or minimize the risk of congenital malformations in the offspring. Beyond hyperglycemia, several factors may also be associated with increased risks of malformations in the offspring of these women, such as obesity, multiple pregnancies, advanced maternal age, folic acid deficiency, use of angiotensin converting enzyme inhibitors and angiotensin receptor blockers, assisted reproduction techniques, and exposure to different types of environmental pollutants.

## INTRODUCTION

According to the World Health Organization (WHO), congenital malformations are abnormalities of structure or function that occur during intrauterine life, that are present from birth or even before, and that can be identified during the prenatal period, at birth, during childhood or even in adulthood (1).

Malformations can be classified based on etiological, clinical and pathogenetic criteria. From an etiological point of view, these anomalies can be classified as primary, due to hereditary causes, and as secondary, which include environmental factors such as chemical or physical agents, metabolic and nutritional conditions, vascular ruptures and mechanical causes (2). Thus, anomalies may have a single cause, be multifactorial or even have no identifiable cause. From a clinical point of view, they can be classified into major or minor. Major malformations are serious anatomical, aesthetic and functional changes that, in general, require intervention, such as neural tube defects and orofacial cleft. Minor malformations are alterations that do not cause serious complications affecting the maintenance of life, such as clinodactyly and bifid tongue (3). From a pathogenetic perspective, malformations are classified into syndromes, when a single etiological factor triggers several structural defects, associated, in which there are different conditions that do not correlate with each other, and dysplasias, when there is an abnormal morphology of specific tissues (2).

Due to the increased life expectancy of people born with congenital malformations and the relationship of most stillbirths and neonatal mortality with malformations (4), this topic has gained increasing attention and importance in public health. Thus, surveillance and notification programs are important tools for monitoring the prevalence and analyzing data to identify possible causes and consequences of congenital malformations, in addition to providing data to refer, structure and evaluate prevention and treatment programs (5).

Currently, there are large networks of information exchange to disseminate these findings, with the most relevant being the Latin American Network of Congenital Malformation Surveillance (ReLAMC), the Global Health Observatory (GHO), the European Surveillance of Congenital Anomalies (EUROCAT) and the International Clearinghouse of Birth Defects Surveillance and Research (ICBDSR) (5).

The newly founded ReLAMC integrates data from hospital and population databases regarding national and regional data in Latin American countries. This system has ten programs: five national, four regional and one international, the ECLAMC (Latin American Collaborative Study of Congenital Malformations) (5). GHO is a WHO-linked service and consists of the most comprehensive and up-to-date online repository of global health data, with free public access. Their data come from government birth and death records, health systems, surveys, censuses, research projects and databases maintained by other organizations (6). ICBDSR, another WHO-affiliated organization created in 1974, associates research programs on congenital malformations with surveillance data from 42 sources (including ECLAMC) located in 36 countries, thus monitoring more than 4 million births annually. Finally, EUROCAT is an European network for epidemiological surveillance of congenital anomalies and comparison between population groups or regions that has existed since 1979. It encompasses population-based data from approximately 750,000 cases of birth defects in 23 countries, covering nearly 30% of European births (one million and seven hundred births per year) (7).

The incidence of major congenital malformations in the offspring of the background population is approximately 3% (2.58% to 3.3%) (8). Among children born to mothers with diabetes mellitus (DM), this rate ranges from 6% to 10% (9). An increase in these rates is expected to occur according to the International Diabetes Federation projections for the overall prevalence of 342.5 million or 10.8% of women with diabetes in 2045. Currently, one in six live births (20 million babies) are born to mothers who have some degree of hyperglycemia during the gestational period, with 84% of the cases being due to gestational diabetes mellitus (GDM) (10). Hyperglycemia is an important teratogenic factor, especially if occurring during the organogenesis period.

Taking into consideration recent or relevant data on this topic, it is necessary to consider the limitation of studies on malformations associated with DM due to the diversity of etiological factors that may be involved, the difficulty of identification and the underreporting of cases (9).

Considering the importance of recognizing congenital anomalies associated with pregnancies complicated by DM and the presence of other factors beyond hyperglycemia in the etiopathogenesis of malformations, this review aims to analyze congenital malformations described in children born to women with hyperglycemia during pregnancy and their pathophysiology.

## MATERIALS AND METHODS

This narrative review addresses the underlying glycemic and nonglycemic mechanisms involved in congenital malformations found in babies born to mothers with hyperglycemia. All the analyzed studies were written in English and Portuguese. Data were collected between April and November 2021, and the searched databases were the National Library of Medicine (PubMed), the Scientific Electronic Library Online (SciELO), and the Global Health Observatory Data Repository. The following descriptors were used: gestational diabetes; hyperglycemia; birth defects; congenital malformations.

### Mechanisms of fetal malformations associated with hyperglycemia

**Figure 1:**
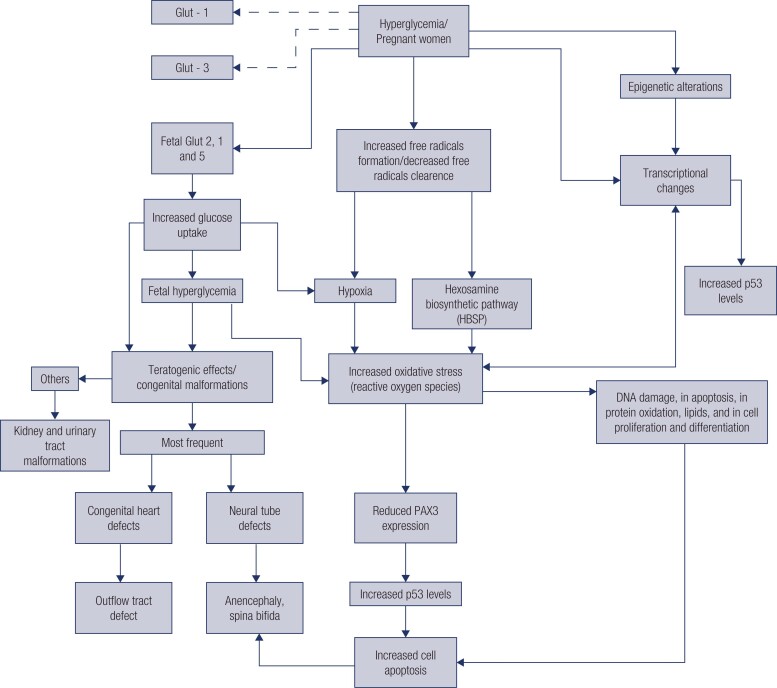
Proposed mechanisms for hyperglycemia-associated malformations.

The mechanisms involved in the development of malformations associated with hyperglycemia in pregnancy are complex (Figure 1).

Glucose transporters (GLUTs) are facilitated diffusion glucose transporters located on cell surfaces that transfer glucose from the extracellular milieu into the cells. There are 14 known isoforms of GLUTs. GLUT-2 has lower glucose affinity than the other GLUTs; consequently, higher glucose levels are required for its activation. In the presence of maternal hyperglycemia, GLUT-2 is overexpressed, which leads to an increased concentration of glucose in the embryonic environment that can ultimately result in fetal malformations (11).

In addition, the lack of GLUT-1 downregulation during a critical period of organogenesis was observed in hyperglycemic rats with digestive system malformations in their offspring. This overexpression of GLUT-1 promotes increased glucose release from the visceral yolk sac into different embryonic cells that are undergoing differentiation processes, which can also lead to malformations (12).

There is also evidence that embryopathies found in pregnant women with DM are related to oxidative stress promoted by hyperglycemia. The relationship between hyperglycemia and the formation of free radicals such as reactive oxygen species can be explained by the occurrence of hypoxia and by the increased flux through the hexosamine biosynthetic pathway (13).

The environment of the newly implanted embryo is hypoxic because there is not a sufficient maternal oxygen supply. In addition, the embryo does not metabolize glucose since there is not an efficient circulatory system and differentiated mitochondrial cells. Thus, a hyperglycemic state associated with hypoxia can result in increased oxidative stress and finally malformations (13).

Regarding the hexosamine biosynthetic pathway, increased glycolytic flux increases the conversion of fructose-6-phosphate and glutamine into glucosamine-6-phosphate (GlcN-6-P), which limits the pentose-phosphate pathway by competition with glucose-6-phosphate to bind glucose-6-phosphate dehydrogenase. The pentose-phosphate pathway inactivates free radicals that induce oxidative stress. When hexosamine biosynthetic pathway activity increases with a subsequent loss of the pentose-phosphate pathway, the elimination of free radicals is impaired. When associated with hypoxia, this causes an increase in oxidative stress (11,13).

Evidence indicates that during the process of embryo formation, oxidative stress participates in the differentiation of stem cells and that changes in this process can alter cell differentiation (14). This explains the relationship between oxidative stress and malformations in children born to mothers with DM, which alters the expression of genes such as *PAX3*, which is directly related to embryo formation. The *PAX3* gene regulates the development of embryonic neuroepithelium and of the somitic mesoderm (11).

Reduced embryonic *PAX3* expression leads to increased expression of the p53 protein, which induces cellular apoptosis and can cause congenital heart malformations, mainly ventricular septation defects (11,15). Congenital heart malformations have multifactorial causes in hyperglycemic women, such as changes in gene expression and chromatin. Some studies suggest that epigenetic modifications may lead to transcriptional changes in target genes associated with maternal hyperglycemia and may cause congenital heart defects (11,16).

Regarding the genitourinary system, excessive amounts of p53 in the metanephric mesenchyme and ureteric bud cell lines can lead to abnormal nephrogenesis resulting from alterations in cell differentiation and apoptosis (17). Genitourinary system malformations can be due to changes in other genes, such as *PAX2* (18).

### Congenital malformations most frequently found among the offspring of mothers with hyperglycemia

Wu and cols. evaluating a large number (29,211,974) of live births to women aged 18 to 49 years registered in the National Vital Statistics System in the US from 2011 to 2018, found an increased risk for congenital malformations in children born to women with preexisting DM (adjusted relative risk (RR): 2.44; 95% CI 2.33-2.55). From a total of cases included in the study, 242,600 were born to mothers with pregestational DM, 1,685,479 to mothers with GDM, and 27,283,895 from euglycemic mothers. A total of 90,061 congenital malformations were reported. The RRs for malformations found in children born to women with preexisting DM, after adjusting for maternal age, race/ethnicity, prepregnancy obesity and infant sex, were 4.61 (95% CI 4.28-4.96) for cyanotic congenital heart disease, 1.88 (95% CI 1.67-2.12) for hypospadias, 2.06 (95% CI 1.82-2.33) for cleft lip with or without cleft palate, 2.35 (95% CI 1.97-2.79) for cleft palate, 1.34 (95% CI 1.10-1.63) for Down syndrome and 2.00 (95% CI 1.59-2.51) for meningomyelocele/spina bifida (9) (Table 1).

**Table 1:** Congenital malformations associated with maternal diabetes

System/Area	Malformation	Authors
Cardiovascular system	Cyanotic congenital heart diseases Ventricular septal defect Patent ductus arteriosus Atrial septal defect Pulmonary artery stenosis Transposition of great vessels Other	Eidem and cols. (22) Chen and cols. (19)
Nervous system	Neural tube defects Anencephaly Microcephaly Isolated hydrocephalus Meningomyelocele/spina bifida Hydrocephalus	Wu and cols. (9) Garne and cols. (20)
Digestive system	Duodenal atresia Anorectal atresia Hypoplastic left colon Gastroschisis Omphalocele	Wu and cols. (9)
Musculoskeletal system	Talipes Arthrogryposis Limb reduction defects Flexion contracture of the limbs Vertebral anomalies	Wu and cols. (9) Eidem and cols. (22)
Urinary system	Kidney malformation Nephron regression	Parimi and Nitsch (21) Eidem and cols. (22)
Orofacial	Orofacial cleft Cleft lip with or without cleft palate Cleft palate alone	Wu and cols. (9)
Caudal regression	Caudal regression syndrome	Garne and cols. (20)
Suspected chromosomal disorder	Down syndrome	Wu and cols. (9)

There is a strong association between maternal DM and congenital heart defects that can show several phenotypes (19). The adjusted prevalence ratio (PR) for congenital heart defects found in the Texas Birth Defects Registry ranged from 1.48-5.28, and the PR for any congenital heart defect was 3.24 (16). In a meta-analysis that included 12 studies from different populations, with 4,207,898 births including 148,869 babies born to mothers with DM and 4,059,027 born to euglycemic mothers, pregestational DM was associated with an RR of 3.59, a 95% confidence interval (CI) of 3.03-4.25 for any congenital heart defect in the offspring. The most prevalent phenotypes were ventricular septal defect (2.09%), atrial septal defect (1.81%), left and right ventricular outflow tract defect (0.31% and 0.45%), pulmonary valve stenosis (0.33%) and coarctation of the aorta (0.18%). A strong association was also found between pregestational DM and truncus arteriosus (RR = 14.49). A Poisson regression was performed considering the following variables: maternal age, race/ethnicity, hypertension, previous live births and smoking (16).

Regarding neural tube defects, the most frequently found malformations associated with maternal DM analyzed by Garne and cols. were anencephaly (adjusted odds ratio [OR]: 1.90 1.20-3.02, p < 0.01) and encephalocele (adjusted OR: 3.27 1.67-6.39, p < 0.01). Caudal regression syndrome is a rare condition that was also strongly associated with maternal DM (adjusted OR = 22.06; CI 6.68-68.72) (20).

Another meta-analysis found that offspring born to mothers with DM had a 50.00% increased risk of congenital abnormalities of the kidney and of the urinary tract compared to offspring born to euglycemic mothers (RR, 1.51; 95% CI 1.36-1.67). Considering only maternal pregestational DM, the risk for congenital abnormalities of the kidney and of the urinary tract almost doubled (RR, 1.97; 95% CI 1.52-2.54). This association persisted after adjustments for maternal body mass index (BMI) (21).

Analysis of all live births between 1999 and 2004 in Norway found that out of 350,961 newborns, 1,583 infants were born to mothers with type 1 diabetes (T1D), showing a prevalence of malformations of 5.70% compared to 2.90% in the background population (adjusted OR: 2.13; 95% CI 1.42-3.20). The variables compared in this study were maternal age, parity, fetal sex, maternal smoking during pregnancy, maternal level of education and race/ethnicity. In more than 50.00% of cases, malformations of the cardiovascular system were reported. In 12.00% of cases, the musculoskeletal system was affected as a single system; in 9.00%, the malformation was restricted to the urogenital system; and in 8.00%, multiple systems were affected (22).

### Glycemic control and reduced risk of malformations

A study conducted in Denmark that included 933 pregnant women with T1D out of 70,089 pregnancies showed that glycated hemoglobin (HbA1c) values higher than 6.90% in the preconception period were associated with a higher risk of congenital malformations. This risk increases continuously with the increase in HbA1c values, and HbA1c values above 10.40% are associated with a 16.00% increase in the risk of fetal malformations (23).

A study that evaluated 2,458 pregnant women with T1D compared to 1,159,865 nondiabetic mothers showed that HbA1c levels found three months before pregnancy and in the first trimester of gestation were associated with a significant and progressive risk of having babies with severe heart malformations. The adjusted hazard ratios for major heart defects in the offspring of women with DM compared to those of women without DM were 2.17 (95% CI 1.37 to 3.42) for HbA1c < 6.5%, 3.17 (95% CI 2.45 to 4.11) for HbA1c between 6.5% and 7.7%, 2.79 (CI 95% 1.90 to 4.12) for HbA1c between 7.8% and 9.0%, and 6.23 (CI 95% 4.32 to 9.00) if HbA1c ≥ 9.1%. The variables considered in this study were year of conception, maternal age, country of birth, living with a partner, education, parity, body mass index, smoking status, and other autoimmune diseases (24).

A meta-analysis that included 25 studies with 5,903 pregnancies showed that preconception care, including improved glycemic control, reduced the risk of congenital malformations by 71.00% (risk ratio 0.29; 95% CI: 0.21-0.40) in children born to women with T1D or type 2 diabetes (T2D) (25).

Another meta-analysis evaluating 12 observational studies with 5,480 mothers with DM showed that poor glycemic control as measured by HbA1c was associated with an increased OR of 3.44 (95% CI, 2.30 to 5.15) for congenital malformations. However, for every 1% decrease in HbA1c values, the relative risk of congenital malformations was reduced by 0.39 to 0.59 (26).

According to the American Diabetes Association and the Brazilian Diabetes Society, women with preexisting DM should be advised to become pregnant when HbA1c values are below 6% or below 7% for those patients with frequent or asymptomatic hypoglycemia (27,28).

### Nonglycemic factors associated with fetal malformations

Beyond hyperglycemia, other risk factors for congenital malformations may be present in pregnancies complicated by DM (Table 2).

**Table 2:** Nonglycemic factors associated with fetal malformations found in children born to mothers with diabetes in pregnancy

Etiology	Malformations that are also associated with hyperglycemia
Alcohol	Congenital heart defects (29) Tetralogy of Fallot Atrioventricular septal defects Conotruncal heart defects
Illicit drugs	Cocaine and methamphetamine (30) Cleft palate and spina bifida Cannabis (30) Anencephaly
Smoking	Cleft lip (with or without cleft palate) (31) Gastroschisis (31) Limb reduction defects (31) Congenital heart defects (31) Renal hypoplasia (32)
Multifetal pregnancies	Gastrointestinal tract anomaly (42) Cardiac anomalies (42)
Advanced maternal age	Congenital heart defects (9,47) Hypospadias (9) Neural tube defects (48) Cleft lip or palate (8,49) Clubfoot (50) Diaphragmatic hernia (9)
Folic acid deficiency	Neural tube defects (51-54) Hydrocephalus (52,53) Cleft lip/palate (51,53) Congenital heart defects (52,54) Conotruncal and septal heart defects (51,54) Limb defects (52,53) Urinary tract anomalies (51-53)
Angiotensin‐converting enzyme (ACE) inhibitors and angiotensin receptor blockers (ARBs)	Cardiovascular defects (60) Central nervous system malformations(60) Urogenital abnormalities (60)
Radiation	Central nervous system malformations (33) Anencephaly (33) Microcephaly (33)
Zika virus	Microcephaly (34) Central nervous system malformation (34)
Assisted reproductive technology (ART): In vitro fertilization (IVF) and Intracytoplasmic sperm injection (ICSI)	Congenital heart defects (66) Nervous system malformations (67)
Environmental pollutants	Tetralogy of Fallot (71,72) Atrial septal defects (72) Pulmonary valve stenosis
Obesity	Neural tube defects (38) Cardiovascular anomalies (38) Cleft palate and cleft lip and palate (38) Limb reduction anomalies (38)

Obesity, multiple pregnancies, advanced maternal or paternal age, folic acid deficiency, use of angiotensin converting enzyme inhibitors (ACE), angiotensin receptor blockers (ARBs), assisted reproduction techniques, and exposure to environmental pollutants are the main factors that can overlap the risk of malformations associated with maternal hyperglycemia. There are also other factors that can contribute to malformations in these pregnancies, such as exposure to alcohol (29), illicit drugs (30), smoking (31,32), radiation (33), and radiation exposure and Zika virus (34).

### Obesity

The worldwide trend of increasing obesity is also affecting women of childbearing age. In the U.S., between 2017 and 2018, 39.7% of women aged 20 to 39 years were obese (35). In 2019, in Brazil, 30.2% of women aged ≥ 20 years had a BMI ≥ 30 kg/m² (36). In Canada, the number of adults with obesity increased from 6.1% to 18.3% between 1985 and 2011. Furthermore, in Australia, approximately 63% of adults, from 2011 to 2012, were overweight and obese (37).

This is a very worrisome reality, since children born to obese mothers have a higher incidence of congenital malformations (38). In addition, the prevalence of obesity is three times higher in patients with T2D than in the general population (39).

### Multiple pregnancies

Multiple pregnancies are also associated with an increased risk of congenital malformations; however, this risk changes according to zygosity and chorionicity (40). In Mexico and Italy, in some registries, an increase of approximately 60% in congenital malformations was found among twins compared to singletons (41).

In Brazil, the prevalence of multiple pregnancies is 1.9% (169 out of 9,000 pregnancies), and of these, 14.2% show congenital malformations (42). The occurrence of GDM is approximately twice as frequent in women with multiple pregnancies as in those with single pregnancies (7.7% vs. 4.1%, respectively) (43). Women with pregestational DM and multiple pregnancies are more likely to have adverse pregnancy outcomes than women with multiple pregnancies without DM (44).

### Advanced maternal age

T2D and GDM are more frequently found in older women (45). Some studies have found a relationship between advanced maternal and paternal ages and increased risk of congenital malformations (46), and some of these malformations are also related to the presence of maternal DM.

Advanced maternal age is directly associated with an increased risk of syndromes that may be associated with congenital heart malformations (47), neural tube defects (48) and cleft lips and palate (49). Some studies have also found an association between advanced maternal age and hypospadias, diaphragmatic hernia (9) and clubfoot (50).

### Folic acid deficiency

Folic acid deficiency (absolute or relative) is a predisposing factor for neural tube defects, which are found in approximately 1/1,000 pregnancies (51-54). Women with pregestational DM or obesity are at higher risk for neural tube defects in their offspring (55). Folic acid supplementation is recommended for all pregnant women and for those planning pregnancy, particularly if they have DM or obesity (56). This risk for neural tube defects could be the result of metabolic alterations found in obesity that can have an impact on folate utilization or increased folate requirements (57) therefore representing a combined risk for the offspring regarding this type of congenital malformation. Nevertheless, to date, there is no conclusive association between DM/obesity and a higher incidence of folic acid deficiency in women.

### Exposure to medications

Exposure to medications accounts for less than 1% of all congenital malformations found in the general population (58). Some drugs are commonly used by women with DM and systemic arterial hypertension, such as angiotensin-converting enzyme inhibitors, angiotensin receptor blockers and statins. The use of angiotensin-converting enzyme inhibitors and angiotensin receptor blockers during pregnancy has been associated with cardiovascular, central nervous system (59) and urinary tract malformations in addition to an increased risk of miscarriage and stillbirth (60).

A recent meta-analysis found that malformations observed on fetuses exposed to such medications during the first trimester of pregnancy do not result from maternal hypertension itself but from these medications, since there were no increased rates of abnormalities in children born to mothers with hypertension treated with other drugs (59,61).

Literature reviews have found no teratogenic effects of metformin in animal models (62). However, more recent studies using mice have shown that metformin can affect early embryonic development, especially at higher doses. (63). No robust data regarding fetal malformations in humans exposed to metformin have been described thus far.

### Assisted reproductive techniques: in vitro fertilization and intracytoplasmic sperm injection

It is estimated that in the US and Europe, the percentage of births after the use of assisted reproductive techniques is 1% and 4%, respectively, whereas in Brazil, this rate is approximately 0.4% (approximately 10 thousand births/year) (64,65). In a meta-analysis and systematic review evaluating 41 studies, which included 25,856 babies born to single or multiple pregnancies resulting from assisted reproductive techniques and 287,995 babies born after spontaneous conception, congenital heart malformations were found in 1.3% and 0.68% of babies, respectively (66). Preliminary studies have found that assisted single pregnancies are associated with a higher risk of malformations, especially in the cardiac and nervous systems, than single spontaneous pregnancies (67). This may occur due to couples’ fertility problems and not due to the assisted reproductive techniques themselves. There was no difference in the risk of malformations between children conceived with in vitro fertilization and those conceived via intracytoplasmic sperm injection (67). Women with DM more frequently present reproductive dysfunction and are generally older (45). Although these factors may be associated, the direct relationship between DM and higher rates of assisted pregnancies has not yet been well established (68).

### Environmental pollutants

An association between exposure to some environmental pollutants and DM has been reported (69,70). Lipophilic products such as polychlorinated biphenyls, dioxins, organochlorine pesticides and brominated flame retardants tend to accumulate in the food chain and can impair human body homeostasis and health. An increased risk for DM has been reported among Vietnamese people exposed to the dioxin-contaminated agent orange (70). The prevalence of congenital malformations varies according to each type of pollutant (71). Three meta-analyses and systematic reviews that evaluated the effect of these environmental pollutants on congenital malformations have found that exposure to carbon monoxide was associated with higher incidence of tetralogy of Fallot; to PM10 (particles less than 10 micrometers in diameter) and ozone, higher incidence of atrial septal defects; to nitrogen dioxide, increased incidence of coarctation of the aorta and pulmonary valve stenosis; and to sulfur dioxide, higher incidence of ventricular septal defects (71,72).

### Limitations

This study has some limitations that must be mentioned. First, we did not have information to distinguish GDM, T1D and T2D in many studies. Second, it is possible that in some women, newly diagnosed overt diabetes could have been misclassified as GDM. Third, several networks of information exchange regarding malformations collect information only at the time of birth. Consequently, some types of congenital anomalies that are identified only in childhood or adulthood would not have been identified at that time and consequently missed. Fourth, only live births were included, and congenital anomalies among miscarriages and stillbirths were not counted. Fifth, all analyzed studies were written in English and Portuguese, which may have caused missing some other interesting studies written in other languages that could have changed the results of this review.

In conclusion, the association between maternal hyperglycemia and fetal organogenesis disorders and, consequently, congenital malformations is well established. There is a decreased expression of *PAX3* in the presence of hyperglycemia, which mainly causes heart and nervous system defects, since this gene is essential for neural tube closure. Hyperglycemia-associated embryopathy is related to hyperglycemia-induced oxidative stress. Furthermore, the increase in glucose uptake by GLUT-2 in mothers with DM contributes to the development of embryopathies.

Strict glycemic control must be obtained before and during pregnancy to prevent fetal malformations. Beyond hyperglycemia, several factors may be associated with increased risks of malformations in women with DM. More studies are warranted to better understand the pathophysiological mechanisms involved in DM-related congenital malformations, aiming to establish appropriate interventions at the appropriate time to reduce the occurrence of these malformations in children born to mothers with hyperglycemia.
